# Squamous Cell Carcinomas of the Skin Explore Angiogenesis-Independent Mechanisms of Tumour Vascularization

**DOI:** 10.1155/2014/651501

**Published:** 2014-05-06

**Authors:** Ievgenia Pastushenko, Tamara Gracia-Cazaña, Sandra Vicente-Arregui, Gert G. Van den Eynden, Mariano Ara, Peter B. Vermeulen, Franciso José Carapeto, Steven J. Van Laere

**Affiliations:** ^1^Department of Dermatology, University Hospital “Clinico Lozano Blesa,” Calle San Juan Bosco 15, 50009 Zaragoza, Spain; ^2^Department of Pathology, University Hospital “Miguel Servet,” Paseo Isabel la Católica 1-3, 50009 Zaragoza, Spain; ^3^Translational Cancer Research Unit Antwerp, Oncology Centre, General Hospital Sint-Augustinus, Oosterveldlaan 24, 2610 Wilrijk, Belgium; ^4^Department of Medicine, Psychiatry and Dermatology, School of Medicine, University of Zaragoza, Calle Domingo Miral s/n, 50009 Zaragoza, Spain; ^5^Department of Oncology, KU of Leuven, Herestraat 49, 3000 Leuven, Belgium

## Abstract

*Aims.* To evaluate the vascularization in basal cell carcinomas (BCCs) and squamous cell carcinomas (SCCs) of the skin. *Methods.* We performed CD31 (i.e., panendothelial marker) and CD105 (i.e., proliferating endothelium marker) immunostaining on samples of 70 SCCs and 70 BCCs of the skin. We evaluated the relative blood vessel area using the Chalkley counting method in each histologic subtype of these tumours. We calculated the degree of proliferation of blood vessel endothelium dividing CD105-Chalkley score by CD31-Chalkley score. *Results.* We found significantly higher peritumoral and intratumoral blood vessel area in SCC when compared to BCC (both with CD31 and CD105). Chalkley counts differed significantly between groups with different BCC histologic subtypes and SCC with different grade of differentiation. Surprisingly, the degree of proliferation of blood vessel endothelium was higher in BCC when compared to SCC. *Conclusions.* While SCC exhibited significantly higher intratumoral and peritumoral blood vessel areas compared to BCC, the relatively low rate of proliferating endothelium in this tumour type suggests the existence of endothelial-sprouting-independent mechanisms of vascularization in SCC.

## 1. Introduction

Basal cell carcinoma (BCC) and squamous cell carcinoma (SCC) of the skin are the most common human solid malignant tumours. One of the key biological processes that appear to be relevant to the establishment and prognosis of most forms of cancer, including skin cancer, is angiogenesis [1]. Using the mouse model Eichten et al. demonstrated that during squamous skin carcinogenesis blood vessels undergo increase in endothelial cell proliferation, vessel diameter, vessel density and leakage [2]. Although several studies evaluated the role of microvessel density in the progression of epithelial skin cancer, the proliferation of vascular endothelium or blood vessel area (BVA) has not yet been investigated.

For many years, the process of tumour vascularization was thought to proceed mainly through the sprouting of new blood vessels from the preexisting vasculature. However, in recent years, additional endothelial-sprouting-independent mechanisms have been described, such as vascular cooption, intussusceptive angiogenesis, mosaic vessels, vasculogenesis, and/or vasculogenic mimicry [3].

The quantification of angiogenesis is complicated by the fact this is dynamic process. Two international consensus on quantification of angiogenesis in solid tumours were published [4, 5], considering the Chalkley counting technique as a more reproducible and clinically applicable technique. Chalkley counting score reflects the relative vessel area, gibing an estimate of the total perfusion of the tumor. It is important to note that two categories of endothelial cell specific antibodies are currently available for immunohistochemistry: the panendothelial cell markers, characterized by equal intensity of staining for small and large blood vessels, and antibodies that bind selectively to proliferating endothelium [6]. In order to evaluate the degree of proliferating blood vessels we used the proliferating-endothelium marker CD105 and we compared the results with those obtained with CD31 immunostain.

## 2. Materials and Methods

### 2.1. Patients and Tissue Samples

A total of 70 formalin-fixed, paraffin-embedded surgical specimens of patients presenting with SCCs and 70 with BCCs at the University Hospital “Clínico Lozano Blesa” (Zaragoza, Spain) between 2008 and 2010 were included in our study. The following clinicopathologic variables were recorded: sex, date of birth, date of diagnosis, anatomic site of the tumor, BCC histologic subtype (superficial, nodular, and aggressive), SCC histological grading (well-, moderately, and poorly differentiated), lymphovascular invasion, and perineural invasion. In the “aggressive” BCC group, we included infiltrative, morpheaform, and micronodular histotypes.

### 2.2. Immunohistochemistry

Immunohistochemical stainings were performed using DAKO autostainer system and commercially available endothelial markers CD31 (monoclonal mouse anti-human platelet endothelial cell adhesion molecule- (PECAM-) 1 antibody, Clone JC70A, DAKO, Glostrup, Denmark; dilution: 1 : 50 for 1 hour) and CD105 (monoclonal mouse anti-human endoglin antibody, Clone SN6h, DAKO, Glostrup, Denmark; dilution: 1 : 100 for 1 hour). As positive control, a sample of breast cancer with a known high MVD was used.

### 2.3. Evaluation of Relative Blood Vessel Area and Proliferating Endothelium

The relative blood vessel area was determined both in intratumoral (IT) and peritumoral (PT) areas using Chalkley counting technique. PT vessels were defined as CD34 positive vessels located at the interface of tumour and adjacent dermis (Chalkley method is based on the use of an eyepiece graticule containing 25 randomly positioned dots, which is rotated so that the maximum number of points is on or within the vessels). Thus, the Chalkley count is the number of points that hit stained vessels. The average of three highest values for each tumor section was used for the analysis.

### 2.4. Statistical Analysis

All analyses were performed using the statistical package SPSS ver. 19.0 for Mac. Associations between different categorical variables were assessed by Pearson's *χ* ^2^ test. The unpaired Student's *t*-test was used variables following normal distribution. The CD31 and CD105 Chalkley counts did not follow the normal distribution (Kolmogorov-Smirnov = 0.12, *P* < 0.0001, and Kolmogorov-Smirnov = 0.10, *P* = 0.002, resp.); therefore, the Mann-Whitney test, Wilcoxon signed-rank test, or Spearman's rank correlation was used as appropriate. The intraobserver and interobserver agreement for classifying the tumours as Chalkley count high or low (using as a cut-off point the mean Chalkley count value) were evaluated by kappa statistic.

## 3. Results

The median age of included patients was 77.6 years (range 60.2–88.3) in SCC group and 75.5 years (range 53.5–88.9) in BCC group at the time of surgery (*P* < 0.05). The main clinical and pathologic characteristics of patients included in the study are compiled in Table 1.

**Figure 1:**
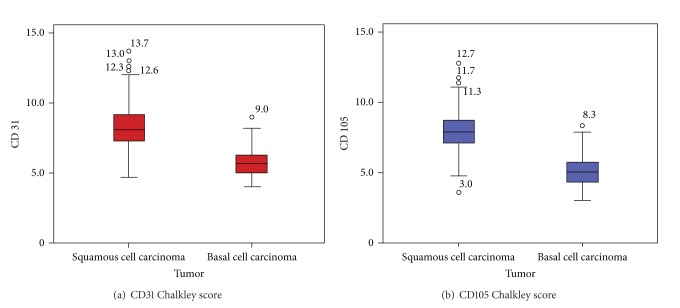
CD31 and CD105 Chalkley counts in basal and squamous cell carcinomas. Box plots for the Chalkley score for CD31 (a) and CD105 (b).

**Figure 2:**
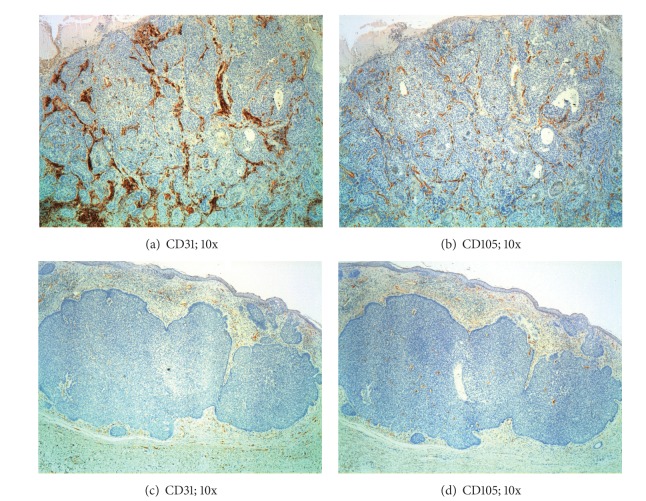
Blood vessels in basal cell carcinoma and squamous cell carcinomas. Sections of squamous cell carcinoma ((a) and (b)) showing a notably higher vessel density than basal cell carcinoma, both for CD31 (c) and CD105 (d).

In all histological slides CD31 or CD105 positive staining could be detected. The costaining of inflammatory cells has been observed in slides immunostained with CD31, but these could be distinguished from endothelial cells on the basis of morphological differences. No clear hot spots were observed in SCC or in BCC. Median peritumoural- (PT-) CD31-Chalkley count was 8.33 (SD = 1.98) points for SCC and 5.81 (SD = 1.05) for BCC (*P* < 0.001) (Figure 1(a)), while median PT-CD105-Chalkley count was 7.84 (SD = 1.76) points for SCC and 5.19 (SD = 1.19) for BCC (*P* < 0.001) (Figure 1(b)). Median intratumoral- (IT-) CD31-Chalkley count was 4.67 (SD = 1.23) for SCC and 0.00 (SD = 1.67) for BCC (*P* < 0.001), while median IT-CD105-Chalkley count was 2.33 (SD = 1.71) and median IT-CD105-Chalkley count was 0.00 (SD = 0.67) (*P* > 0.001). Moreover, both PT and IT-rBVA differed between superficial, nodular, and aggressive BCC (*P*_CD31_ < 0.001, *P*_CD105_ < 0.001) and between well-, moderately, and poorly differentiated SCC (*P*_CD31_ = 0.007, *P*_CD105_ = 0.03) (Table 2).  Figure 2 shows samples of CD31 and CD105 immunostaining in BCC and SCC. There was a strong correlation between CD31 and CD105 Chalkley score (Pearson's *R* = 0.8; *P* < 0.001). We next proceeded to evaluate the degree of proliferating endothelium by dividing the CD105-Chalkley score by the CD31-Chalkley score. The mean CD105/CD31 quotient was 0.96 for BCC (SD = 0.16) and 0.79 for SCC (SD = 0.16) (*P* = 0.024).

In a subset of cases (*n* = 30; 15 SCCs and 15 BCCs), the intraobserver and the interobserver agreement were evaluated by kappa statistic (both for CD31 and CD105). These slides were examined by IP two times and one time by TGC. The intraobserver agreement between the two examinations by IP for classifying the tumors as high or low Chalkley counting values (taking the mean value for each antibody as a cut-point) was *k* = 0.65 for CD31 and *k* = 0.80 for CD105, whereas the interobserver agreement between IP and TGC was *k* = 0.54 for CD34 and *k* = 0.67 for CD105 (*k*-values between 0.41 and 0.60 indicate moderate strength of agreement and *k*-values between 0.61 and 0.80-substantial strength of agreement).

## 4. Discussion

In our present study, we found statistically greater relative PT and IT blood vessel areas in SCC compared to BCC, both with CD31 and CD105 antibodies. These findings seem to be logical, since high degree of vascularization provides oxygen and nutrients for tumour growth, explaining, at least in part, the differences in clinical behavior between BCC and SCC. While most studies evaluating the role of angiogenesis in cancer, including epithelial skin tumours, used the technique described by Weidner et al., based on the number of microvessels quantification, we evaluated the blood vessel area, reflecting the global tumour perfusion. Loggini et al. investigated the angiogenesis pattern in BCC and SCC of the skin, demonstrating that the majority of samples with high vascularization belonged to the squamous histotype, while the majority of BCC showed a significantly lower number of microvessels [7]. Chin et al. evaluated vascular patterns in SCCs, BCCs, and trichoepitheliomas (TEs) using CD31 mAb. The SCC blood vessel counts were significantly higher if compared to BCCs or TEs [8]. Differences between the neovascularization in BCC with benign course and BCC with aggressive behavior have been found [9, 10]. From the clinical perspective, the phase II clinical trial based on 31 patients with advanced cutaneous SCC treated with Cetuximab showed a disease control rate in 69% and response rate in 28% of included patients [11].

However and surprisingly, BCC exhibited significantly higher proportion of proliferating endothelium than SCC, apparently contradicting the abovementioned results. There is at least one explanation for these findings, based on angiogenesis-independent mechanisms of tumour vascularization. Intussusceptive angiogenesis occurs by the insertion of the interstitial tissue column into the lumen of preexisting vessels inducing partition of the vessel lumen [3]. This type of angiogenesis is faster and more economical than sprouting, occurs within hours of even minutes, and does not primarily depend on EC proliferation. The ability for growing by coopting the massive vascular plexus present in the peritumoural connective tissue was described in aggressive tumours localized in highly vascularized tissues. Thus, our results suggest that SCC, characterized by more rapid growth and aggressive clinical behaviour, explores other mechanisms of tumour vascularization which allow faster oxygen and nutrient transport, while BCC growth mainly depends on proliferation of vascular endothelium. Importantly, angiogenesis-independent mechanisms of tumour vascularization were described to be more resistant to antiangiogenic therapy.

Angiogenesis is a dynamic process, making its quantification inherently difficult. Since no decisions have to be made on whether adjacent stained structures are the reflection of one single microvessel or two separate microvessels, Chalkley counting is considered a more objective approach than Weidner technique [5]. We evaluated the reproducibility of this technique, showing both inter- and intraobserver moderate to substantial strength of agreement. Slightly lower agreement in CD31-immunostained slides could be explained by the presence of costaining of inflammatory cells, which could result in the erroneous incorporation of inflammatory cell count into the final score.

In conclusion, our results suggest that the existence of angiogenesis-independent mechanisms of tumour vascularization may be considered as a hallmark of epithelial tumors with more aggressive clinical behaviour.

## Figures and Tables

**Table 1:** Summary of the main clinical and histological characteristics of patients included in the study.

Category	BCC	SCC
Number of patients		
Male	53	49
Female	17	21
Total	**70**	**70**
Age of diagnosis	73.3 (53.5–88.9)	76.2 (60.2–88.3)
Anatomic site		
Head and neck	51 (72.8%)	53 (75.7%)
Extremities	6 (8.6%)	15 (21.4%)
Trunk	13 (18.6%)	2 (2.9%)
Histologic subtype	Superficial BCC: 23 (32.9%)	Well-differentiated SCC: 30 (42.8%)
	Nodular BCC: 42 (60.0%)	Moderately differentiated SCC: 37 (52.9%)
	Aggressive* BCC: 5 (7.1%)	Poorly differentiated SCC: 3 (4.3%)
Presence of LVI	0 (0%)	3 (4.3%)
Presence of PNI	0 (0%)	1 (1.4%)

BCC: basal cell carcinoma; SCC: squamous cell carcinoma; LVI: lymphovascular invasion; PNI: perineural invasion.

*Aggressive BCC group includes infiltrative, morpheaform, and micronodular subtypes.

**Table 2:** Chalkley score according to histotype in basal and squamous cell carcinomas.

CD31			K-W	CD105			K-W
Basal cell carcinoma							
Superficial (*n* = 23)	Nodular (*n* = 42)	Aggressive (*n* = 5)	*P* < 0.001	Superficial (*n* = 23)	Nodular (*n* = 42)	Aggressive (*n* = 5)	*P* < 0.001
5.2 (SD = 0.64)	6.0 (SD = 0.94)	7.2 (SD = 1.17)		4.2 (SD = 0.73)	5.4 (SD = 0.86)	7.7 (SD = 0.69)	

Squamous cell carcinoma							
Well-diff. (*n* = 30)	Moderately diff. (*n* = 37)	Poorly diff. (*n* = 3)	*P* = 0.07	Well-diff. (*n* = 30)	Moderately diff. (*n* = 37)	Poorly diff. (*n* = 3)	*P* = 0.03
7.7 (SD = 1.43)	8.7 (SD = 1.91)	12.1 (SD = 2.14)		7.3 (SD = 1.53)	8.2 (SD = 1.54)	11.4 (SD = 1.2)	

The CD31 and CD105 Chalkley counts did not follow the normal distribution (Kolmogorov-Smirnov = 0.12, *P* < 0.0001, and Kolmogorov-Smirnov = 0.10, *P* = 0.002, resp.). Therefore, K-W (=Kruskal-Wallis) test has been applied.
